# Differential activity of innate defense antimicrobial peptides against *Nocardia *species

**DOI:** 10.1186/1471-2180-10-61

**Published:** 2010-02-23

**Authors:** Siegbert Rieg, Benjamin Meier, Eva Fähnrich, Anja Huth, Dirk Wagner, Winfried V Kern, Hubert Kalbacher

**Affiliations:** 1 Center for Infectious Diseases & Travel Medicine, University Medical Center, Hugstetter Strasse 55, 79106 Freiburg, Germany; 2 Medical and Natural Sciences Research Center, University of Tübingen, Ob dem Himmelreich 7, 72074 Tübingen, Germany

## Abstract

**Background:**

Members of the genus *Nocardia *are ubiquitous environmental saprophytes capable to cause human pulmonary, disseminated and cutaneous nocardiosis or bovine mastitis. Innate immunity appears to play an important role in early defense against *Nocardia *species. To elucidate the contribution of antimicrobial peptides (AMPs) in innate defense against *Nocardia*, the activity of human α-defensins human neutrophil peptides (HNPs) 1-3, human β-defensin (hBD)-3 and cathelicidin LL-37 as well as bovine β-defensins lingual and tracheal antimicrobial peptides (LAP, TAP) and bovine neutrophil-derived indolicidin against four important *Nocardia *species was investigated.

**Results:**

Whereas *N. farcinica *ATCC 3318 and *N. nova *ATCC 33726 were found to be susceptible to all investigated human and bovine AMPs, *N. asteroides *ATCC 19247 was killed exclusively by neutrophil-derived human α-defensins HNP 1-3 and bovine indolicidin. *N. brasiliensis *ATCC 19296 was found to exhibit complete resistance to investigated human AMPs and to be susceptible only to bovine indolicidin.

**Conclusion:**

Selected AMPs are capable to contribute to the first line of defense against *Nocardia*, yet, susceptibility appears to vary across different *Nocardia *species. Obtained results of neutrophil-derived AMPs to possess the broadest antinocardial spectrum are remarkable, since nocardiosis is characterized by a neutrophil-rich infiltrate *in vivo*.

## Background

*Nocardia *represent a genus of aerobic actinomycetes and belong specifically to the family *Mycobacteriaceae *[1]. *Nocardia *are aerobic, gram-positive, filamentous, branching rods and can be found as ubiquitous environmental saprophytes in soil, dust, organic matter and water. Due to recent advances in phenotypic and molecular characterization (especially 16S rRNA gene sequencing) the spectrum of *Nocardia *has expanded, with more than 30 species described [2]. At least 13 *Nocardia *species have been implicated in human infection with varying geographic prevalence throughout the world [3].

Human infections usually arise from inhalation or direct inoculation into skin or soft tissue structures. Major forms of *Nocardia *infection are pulmonary nocardiosis, disseminated and CNS nocardiosis, cutaneous/lymphocutaneous nocardiosis and mycetoma. Nocardiosis may be considered as opportunistic infection with chronic lung disease (often in association with long-term corticosteroid treatment), transplantation, malignancies, diabetes mellitus and alcohol abuse as most prevalent underlying conditions [4]. Nevertheless, a review of more than 1000 cases of *Nocardia *infection revealed no identifiable predisposing immunocompromising factors in approximately 30% of patients [5]. Additionally, *Nocardia *are well-recognized pathogens in animals with bovine masititis representing the most important infection.

The characteristic histopathological feature of nocardiosis consisting of an acute pyogenic inflammatory reaction i.e. a predominant neutrophil-rich infiltrate as well as results of prior studies point towards an important role of innate defense mechanism against *Nocardia *species.

Antimicrobial peptides (AMPs) represent evolutionarily conserved multifunctional molecules of innate immunity. In mammals, AMPs like human β-defensins (hBD) 1-3 and bovine lingual or tracheal antimicrobial peptide (LAP, TAP) are expressed by cells within the epithelial lining or are delivered to sites of infection by circulating leukocytes [6-8]. Examples of the latter group of AMPs include human neutrophil peptides (HNPs) 1-3, bovine indolicidin or human cathelicidin LL-37 [9-11]. AMPs are produced constitutively or are induced upon infection or inflammation and exert activity against a broad spectrum of microorganisms including gram-positive and gram-negative bacteria, enveloped viruses, protozoa and fungi [12]. Apart from a direct microbicidal effect, AMPs exhibit a variety of additional functions by promoting chemotaxis and phagocytosis, stimulating angiogenesis and wound healing or neutralizing LPS effects [13].

There is increasing evidence that AMPs take part in innate defense against Nocardia-related bacteria such as *Mycobacterium tuberculosis *[14] or *Actinomyces israelii *and *naeslundii *[15]. The aim of the current study was to elucidate the contribution of AMPs in innate immunity against different *Nocardia *species. We therefore investigated the activity of several important epithelial- and neutrophil-derived human and bovine AMPs against the four nocardial species *N. farcinica*, *N. nova*, *N. asteroides *and *N. brasiliensis*, all of whichrepresent major human and bovine pathogens.

## Results and Discussion

Levofloxacin was used as killing control to compare antinocardial potency of tested AMPs and showed dose-dependent activity against all four nocardial strains. The peptide DPY without antimicrobial activity served as negative control and exhibited no activity against all tested *Nocardia *strains (data not shown).

### Activity of human AMPs against Nocardia species

**Figure 1:**
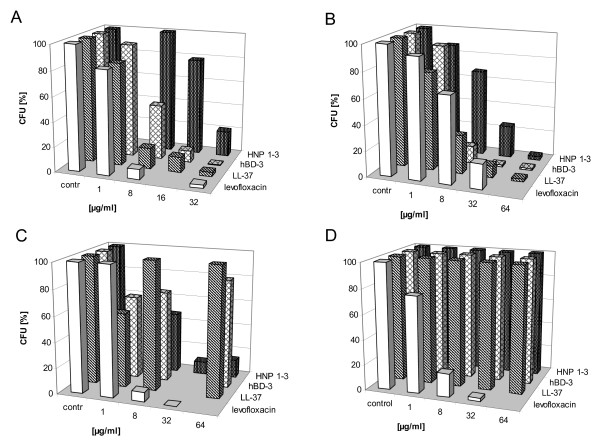
**Activity of human AMPs HNP 1-3, hBD-3, LL-37 and levofloxacin (killing control) against A *N***. *farcinca *ATCC 3318 (p < 0.05 for all tested substances), **B ***N*. *nova *ATCC 33726 (p < 0.05 for all tested substances), **C ***N*. *asteroides *ATCC 19247 (levofloxacin p < 0.05, HNP1-3 p = 0.11) and **D ***N*. *brasiliensis *ATCC 19296 (levofloxacin p < 0.05) was investigated using a colony forming unit (CFU) assay. Data are means (percent CFU reduction) of at least four independent sets of experiments with each peptide and each *Nocardia *species.

All tested human AMPs exhibited activity against *N. farcinica *ATCC 3318 (Figure 1A) and *N. nova *ATCC 33726 (Figure 1B). Human β-defensin hBD-3 revealed strongest activity with LD_90 _of 16 μg/ml against both strains. Human cathelicidin LL-37 showed LD_90 _of 32 μg/ml respectively. Accordingly, we found human α-defensins HNP 1-3 to be active, although higher concentrations were needed with LD_90 _>32 μg/ml against *N. farcinica *and LD_90 _of 64 μg/ml against *N. nova *(Table 1). Notably, hBD-3 and LL-37 were found to be more potent against *N. nova *than levofloxacin in equivalent concentrations.

**Table 1:** Susceptibility of different *Nocardia *species against innate defense AMPs

	**LD**_**90**_**(μg/ml)** (killing/CFU reduction in percent ± SD)						
	
Species	levoflox	HNP 1-3	LL-37	hBD-3	indolicidin	LAP	TAP
*N. farcinica *ATCC 3318	8 (92.3 ± 3.8)	>32	32 (96.6 ± 0.6)	16 (92.5 ± 5.3)	16 (96.7 ± 1.7)	16 (92.9 ± 7.1)	32 (94 ± 5.1)
*N. nova *ATCC 33726	>32	64 (97.2 ± 3.6)	32 (91.4 ± 7.0)	16 (95.2 ± 1.7)	8 (90.5 ± 3.4)	n.d.	n.d.
*N. asteroides *ATCC 19247	8 (92.6 ± 3.8)	32 (90.9 ± 0.6)	>64	>64	64 (99.1 ± 0.6)	n.d.	n.d.
*N. brasiliensis *ATCC 19296	32 (96.6 ± 2.2)	>64	>64	>64	64 (92.9 ± 2.1)	>64	>64

LD_90 _denotes the lowest peptide concentration leading to a = 90% reduction of CFU after incubation (12 h or 16 h) with AMPs or levofloxacin. Presented data are LD_90 _determinations based on means of at least four (levofloxacin, HNP 1-3, LL-37 and hBD-3) or two (indolicidin, LAP and TAP) independent sets of experiments with each *Nocardia *species. In brackets are means (percent CFU reduction) ± standard deviation of respective experiments.

SD standard deviation, n.d. not determined

**Figure 2:**
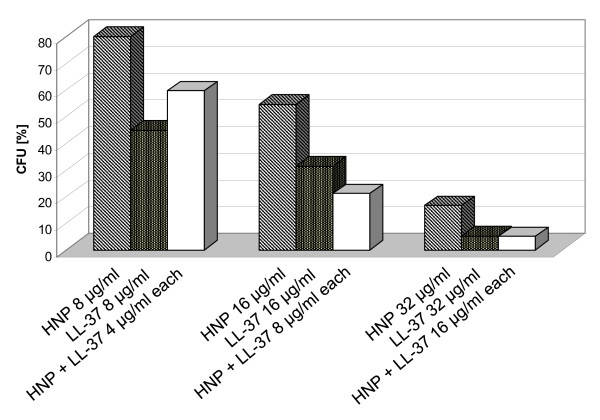
**Additive activity of the two AMPs HNP 1-3 and LL-37 in a colony forming unit (CFU) assay against *N*. *farcinica *ATCC 3318**. A combination of HNP 1-3 and LL-37 exhibited killing comparable to each peptide alone at twofold higher concentrations (i.e. 78.9% CFU reduction by 8 μg/ml HNP 1-3 in combination with 8 μg/ml LL-37 compared to 68.5% CFU reduction by 16 μg/ml LL-37 or 45.6% reduction by 16 μg/ml HNP 1-3 alone). Data are results of a single assay.

To address additive or synergistic effects of AMPs, we performed a model assay using *N. farcinica *and a combination of LL-37 and HNP 1-3 (Figure 2). Since the combination of the two AMPs exhibited nocardial killing comparable to each peptide alone at twofold higher concentrations, we found additive activity of the two AMPs.

In contrast to results with *N. farcinica *and *N. nova*, hBD-3 and LL-37 did not show antinocardial activity against *N. asteroides *ATCC 19247 (Figure 1C). Only human α-defensins HNP 1-3 were found to be active against *N. asteroides *with LD_90 _of 32 μg/ml.

*N. brasiliensis *ATCC 19296 proved to be resistant to all human AMPs tested since neither HNP 1-3 nor hBD-3 or LL-37 exhibited killing activity in concentrations up to 64 μg/ml (Figure 1D). Remarkably, stronger growth of *N. brasiliensis *was observed with all three AMPs investigated. Enhanced growth was not found after incubation with equivalent concentrations of DPY (data not shown). To investigate whether proteolytic degradation of AMPs by *N. brasiliensis*-derived proteases might play a role, we added a protease inhibitor mix during incubation in CFU assays. Protease inhibitors were not able to alter the observed AMP resistance of *N. brasiliensis*, yet enhanced growth of *N. brasiliensis *after co-incubation with protease inhibitors could be observed again(data not shown).

### Activity of bovine AMPs against Nocardia species

**Figure 3:**
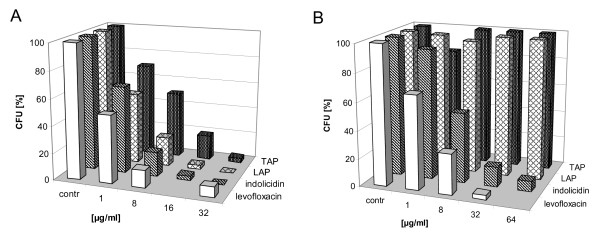
**Activity of bovine AMPs TAP, LAP indolicidin and levofloxacin (killing control) against A *N***. *farcinca *ATCC 3318 (p < 0.05 for all tested substances), **B ***N*. *brasiliensis *ATCC 19296 (indolicidin and levofloxacin p < 0.05) was investigated using a colony forming unit (CFU) assay. Data are means (percent CFU reduction) of at least two independent sets of experiments with each peptide and each *Nocardia *species.

CFU-assays revealed activity of all tested bovine AMPs against *N. farcinica *ATCC 3318 (Figure 3A). Neutrophil-derived indolicidin and bovine β-defensin LAP showed potent killing with LD_90 _of 16 μg/ml respectively. Bovine TAP was also active, LD_90 _proved to be 32 μg/ml. All bovine AMPs revealed at least comparable or greater activity at 32 μg/ml against *N. farcinica *than levofloxacin. Accordingly, bovine indolicidin exhibited killing activity against *N. nova *(LD_90 _8 μg/ml) and *N. asteroides *(LD_90 _64 μg/ml) (Table 1).

In contrast to human AMPs, bovine indolicidin exhibited activity against *N. brasiliensis *ATCC 19296, thus, representing the sole AMP capable to kill *N. brasiliensis *(Figure 3B). LD_90 _of indolicidin was 64 μg/ml. According to results of hBD-3, *N. brasiliensis *proved to be resistant to bovine β-defensins LAP and TAP. Again, pronounced growth was observed after incubation with both AMPs (data not shown).

AMPs are effector molecules of innate immunity and provide a first line of defense against invading pathogens. Our investigations reveal a differential activity of epithelial- and neutrophil-derived AMPs against four members of the genus *Nocardia*. Whereas *N. farcinica *and *N. nova *were found to be susceptible to all investigated human and bovine AMPs, *N. asteroides *was killed exclusively by human α-defensins HNP 1-3 and bovine indolicidin.

Host-pathogen interactions in *Nocardia *species have been extensively studied (for review see Beaman et al.) [5]. Severity and manifestations of nocardiosis are influenced by the portal of entry, tissue tropism, inoculum dose and virulence characteristics of the infecting *Nocardia *strain and, conversely, the efficacy/virtue of the mounted host immune response. Innate defense mechanisms, specifically killing and elimination by neutrophils and macrophages appear to be of particular importance for the outcome of nocardiosis. Although insufficient to resolve infection, the early phagocyte attack is considered to retard infection until lymphocyte-mediated cytotoxicity and activated macrophages accomplish a definite response [16-18].

Constituting a major part of the microbicidal mechanisms of neutrophils, we propose AMPs to contribute to the early phase of defense against various *Nocardia *species. Interestingly and in favour of our hypothesis, we found neutrophil-derived AMPs such as human HNP 1-3 and bovine indolicidin to have broader antinocardial activity than the investigated epithelial AMP hBD-3 (albeit in equimolar concentrations hBD-3 exhibited greater CFU reduction/killing of *N. farcinica *and *N. nova *than HNP1-3). Moreover, besides their abundant presence in neutrophils, AMPs are produced by other innate defense effector cells. LL-37 and, to a lesser extent, HNP 1-3 were found in monocytes/macrophages, NK cells and γδ T cells [19], which are also considered to take part in antinocardial defense.

Several virulence determinants of *Nocardia *including lysozyme resistance and inhibition of phagosome-lysosome fusion have been described [20]. Due to prior investigations, host-pathogen interactions are best charaterized in *N. asteroides *infection. A distinct feature of virulent strains of *N. asteroides *is the capability to resist to oxidative burst-mediated killing by phagocytes due to catalase [21] and superoxide dismutase production [22]. Here we found HNP 1-3 and indolicidin to represent nocardicidal effector molecules belonging to the armament of non-oxidative killing mechanisms of neutrophils. In accordance with our observations, Filice et al. found neutrophil granule-derived cationic proteins (containing HNP 1-3) to inhibit *N. asteroides *growth and filament formation [23].

In human neutrophils, α-defensins HNP 1-3 are stored as active peptides in primary (azurophil) granules in concentrations of >10 mg/ml [24]. As granule-contents are minimally diluted after fusion with the phagocytic vacuole, HNP 1-3 targets ingested pathogens in concentrations multitudes higher than those needed for potent antinocardial killing observed in our study (LD_90 _of *N. farcinca*, *N. nova *and *N. asteroides *= 64 μg/ml). In contrast, the human cathelicidin is stored as inactive precursor hCAP-18 in secondary (specific) granules and is processed to LL-37 by proteinase 3 after secretion into the extracellular milieu. Like the human β-defensin hBD-3, LL-37 is additionally produced upon infection or inflammation by epithelial cells of the respiratory/gastrointestinal tract or by keratinocytes. Levels of LL-37 e.g. in airway surface fluids are estimated to be 1-5 μg/ml [25]. Concentrations of β-defensins are estimated to be in the range of 1-10 μg/ml [13]. Thus, *in vivo *concentrations of LL-37 and hBD-3 will most likely be not sufficient to exert direct nocardial killing. Nevertheless, LL-37 and hBD-3 may take part in antinocardial defense by additive or synergistic action with other antimicrobial peptides and proteins abundantly present along epithelial barriers. In favour of this hypothesis, we found additive killing of *N. farcinica *in a model assay using a combination of LL-37 and HNP 1-3. Moreover, owing to a wide range of biological activities, LL-37 and hBD-3 may further contribute due to chemotactic effects on neutrophils, monocytes and T cells [26,27].

We found *N. brasiliensis *to exhibit complete resistance to all investigated human AMPs and to be susceptible only to bovine indolicidin. *N. brasiliensis *is the most frequently reported cause of progressive cutaneous and lymphocutaneous disease. Furthermore, *N. brasiliensis *often causes infection in otherwise immunocompetent hosts. These clinical features are in accordance with our findings, demonstrating a complete resistance of *N. brasiliensis *against human epithelial, i.e. keratinocyte-derived and neutrophil-derived AMPs.

*N. brasiliensis *is known to produce a variety of proteases [28]. To evaluate a potential resistance due to proteolytic degradation of AMPs (particularly linear α-helical LL-37), CFU-assays were conducted in the presence of protease inhibitors. However, protease inhibitors did not alter AMP-resistance in *N. brasiliensis*. One might speculate about species-specific variances in bacterial cell wall constituents yielding to differential nocardial AMP susceptibility/resistance [29]. Additionally, other mechanisms, i.e. active efflux by multi-drug transporters or modifications on the bacterial cell surface may confer AMP resistance.

The current study revealed *N. brasiliensis *to be susceptible only to indolicidin, a tryptophan- and proline-rich 13 amino acid peptide of bovine neutrophils. In contrast to the predominantly membrane-active human defensins and cathelicidin LL-37, indolicidin has been referred to exert intracellular modes of action with inhibition of DNA and protein-synthesis and alteration of cytoplasmic membrane septum formation [30]. Thus, its distinct chemical features and alternative mode of action may contribute to the unique activity of indolicidin against *N. brasiliensis*.

## Conclusions

Selected AMPs are capable to contribute to the first line of defense against *Nocardia*, yet, susceptibility appears to vary across different *Nocardia *species. Interestingly, our finding of neutrophil-derived AMPs to possess a broad antinocardial spectrum is paralleled by the characteristic feature of a neutrophil-rich infiltrate in histopathological specimens of nocardiosis. Moreover, the observed resistance of *N. brasiliensis *is remarkable, since *N. brasiliensis *is frequently reported to cause cutaneous and lymphocutaneous disease in otherwise immunocompetent hosts. Further studies should address in more detail the differential activity of AMPs, its causes and pathophysiologic significance.

## Methods

### Bacterial strains and culture conditions

Four strains of the genus *Nocardia *were investigated: *Nocardia farcinica *(ATCC 3318), *Nocardia nova *(ATCC 33726), *Nocardia asteroides *(ATCC 19247) and *Nocardia brasiliensis *(ATCC 19296). Strains were grown on Columbia blood agar for at least 72 hours at 37°C. Then 30 ml of Mueller-Hinton-broth (MHB) supplemented with 1% Tween 80 (Serva, Heidelberg, Germany) was inoculated with one loop of bacteria scraped off the agar plates. MHB was incubated in a shake incubator (220 rpm at 37°C). 10 ml of the culture was transferred to a 50 ml tube which contained 1 mm glass beads (BioSpec Products, Bartlesville, USA). After vortexing for 10-15 seconds a homogenous suspension could be gained. A few millilitres of the suspension were used to inoculate another 50 ml of MHB (also supplemented with 1% Tween 80). Cultures were incubated until mid-logarithmic phase was reached. Incubation times were different for each *Nocardia *species (*N. farcinica *12 h, *N. nova *24 h, *N. asteroides *16 h, *N. brasiliensis *72 h).

### Innate defense antimicrobial peptides

The activities of major human and bovine AMPs belonging to different families of AMPs were tested (summarized in Table 2): human cathelicidin LL-37, human α-defensins human neutrophil peptides 1-3 (HNP 1-3) and human β-defensin-3 (hBD-3), bovine indolicidin and bovine β-defensins lingual antimicrobial peptide (LAP) and tracheal antimicrobial peptide (TAP). Human cathelicidin LL-37, bovine indolicidin, LAP and TAP were synthesized using standard Fmoc/tBu chemistry on a multiple peptide synthesizer Syro II (MultiSynTech, Witten, Germany). Oxidation of the reduced LAP and TAP was achieved by dissolving the prepurified peptide with 2 M acetic acid and dilution to a peptide concentration of 0.015 mM in the presence of reduced/oxidized glutathione (molar ratio of peptide/GSH/GSSG was 1:100:10) and 2 M guanidine hydrochloride. The solution was adjusted to pH 8.0 with aqueous NH_4_OH and stirred slowly at 4°C for 3 days. The folding reaction was monitored by analytical HPLC. The solution was concentrated using a C18 Sep-Pak cartridge (Waters, Milford, USA) and lyophilized. Purification of the oxidized products was achieved first by chromatography on a C8 column using the system above and yielding a purity of 90%. Finally, the product was highly purified on a C18 column using a 60 min gradient resulting in a purity of 95%. The quality of the product was confirmed by analytical HPLC, matrix-assisted laser desorption/ionization time of flight mass spectrometry (MALDI-MS), and electrospray ionization mass spectrometry (ESI-MS), yielding the correct mass of oxidized products. Human α-defensins HNP 1-3 were isolated from peripheral neutrophils as previously described [31]. Synthetic hBD-3 was purchased from PeptaNova, Sandhausen, Germany.

**Table 2:** Features of human AMPs used in this study

AMP	class/structure	origin	expression pattern
LL-37	cathelicidin, α-helical peptide	human	neutrophils, monocytes/macrophages (constitutive); epithelial cells of respiratory, gastrointestinal and urogenital tract, keratinocytes (inducible)
HNP 1-3	α-defensins, β-sheet peptides	human	neutrophils (constitutive)
hBD-3	β-defensin, β-sheet peptide	human	epithelial cells of respiratory and gastrointestinal tract, keratinocytes (inducible)
indolicidin	linear, tryptophan- and proline-rich peptide	bovine	neutrophils (constitutive)
LAP	β-defensin, β-sheet peptide	bovine	epithelial cells of respiratory and gastrointestinal tract, mammary gland (inducible)
TAP	β-defensin, β-sheet peptide	bovine	epithelial cells of respiratory tract (inducible)

Levofloxacin (Roussel-Uclaf, Romainville, France) was used as killing control and dissolved in water. A 30 amino acid peptide named DPY without antimicrobial activity was used as negative control [32]. DPY was synthesized using standard F-moc/tBu chemistry and purified by HPLC according to the protocol used for HNP 1-3. All peptides were dissolved in 0.01% acetic acid. Antimicrobial agents were stored at -20°C and were defreezed and freezed three times at a maximum to ensure full antimicrobial activity.

### Colony forming unit assay

A colony forming unit (CFU) assay was established and performed to test AMP susceptibility. Mid-logarithmic growth phase cultures were washed twice in 10 mM sodium phosphate buffer (ph 7.4). A standard inoculum of 1 × 10^7^CFU/ml in 10 mM sodium phosphate buffer supplemented with 1% MHB was prepared. 80 μl of the standard inoculum were incubated with 20 μl of the respective concentrations of the antimicrobial agents in the shake incubator at 37°C for 12 h (*N. farcinica*) to 16 h (*N. nova*, *N. asteroides *and *N. brasiliensis*). Incubation was carried out in 2 ml tubes (Eppendorf, Hamburg, Germany) which were occluded by LidBacs (Eppendorf, Hamburg, Germany) to avoid contamination and provide constant aerobic conditions. After incubation serial dilutions were plated on Mueller-Hinton agar plates and visible colonies were counted after 48-72 hours of incubation at 37°C. Killing was expressed in percentage of bacteria that were killed by incubation with respective peptide concentrations compared to incubation with solvent of the antibacterial substance (0,01% acetic acid or water). LD_90 _denotes the lowest peptide concentration leading to a =90% reduction of CFU counts. CFU assays were at least performed three times and final results are displayed as mean value of all assays. Killing activity (CFU counts after incubation with solvent vs. CFU counts after incubation with highest concentration of AMPs or levofloxacin) was analysed by Student's t-test. A p-value < 0.05 was considered significant.

For testing *N. brasiliensis*, CFU assays were additionally performed by adding a protease inhibitor mix (Complete Mini, Roche, Mannheim, Germany). 10 μl of the protease inhibitor mix were added to the standard inoculum during the 16 h incubation period. Further testing was performed as described above.

## Authors' contributions

SR conceived of the study, drafted and wrote the manuscript and participated in experiments. BM performed antimicrobial assays and helped to draft the manuscript. EF performed antimicrobial assays. AH performed antimicrobial assays. DW participated in the design of the study and analysis of its results. WVK conceived of the study, participated in its design and coordination and edited the manuscript. HK synthesised antimicrobial peptides and helped to draft and edit the manuscript. All authors have read and approved the final manuscript.
